# Adding Behaviour-Change Counselling to an Exercise Program for Adults Preparing for Hip and Knee Arthroplasty Improves Psychological and Physical Wellness: Focus Group Reflections

**DOI:** 10.3390/ijerph20206960

**Published:** 2023-10-23

**Authors:** Marie-Louise Bird, Jonathan Mulford, Andrew Daffyd Williams, Michael Cheney, Jane O’Brien

**Affiliations:** 1 School of Health Sciences, University of Tasmania, Launceston, TAS 7250, Australia; andrew.williams@utas.edu.au (A.D.W.); michael.cheney@utas.edu.au (M.C.); 2 Department of Physical Therapy, University of British Columbia, Vancouver, BC V6T 1Z3, Canada; 3 Launceston General Hospital, Launceston, TAS 7250, Australia; jonathanmulford1971@gmail.com; 4 School of Nursing and Midwifery, University of Tasmania, Launceston, TAS 7250, Australia; j3.obrien@qut.edu.au; 5 School of Nursing, Queensland University of Technology, Brisbane, QLD 4059, Australia

**Keywords:** clinical rehabilitation, hip and knee waitlist, osteoarthritis, physical exercise, benefits

## Abstract

Purpose: To explore participant experiences for people on an arthroplasty waitlist, randomised to an exercise and behaviour-change counselling program (ENHANCE). The ENHANCE program for arthroplasty patients was led by an accredited exercise physiologist who delivered an individually tailored and structured exercise program. Included in the exercise program were up to five in-person counselling sessions, based on the Health Action Process Approach (HAPA) applied specifically to people with osteoarthritis. Nine adults (mean 69.4 years) who were on the waiting list for a total hip or knee arthroplasty and who had completed a 12-week program (ENHANCE) as part of a randomised controlled trial were recruited for this study. Methods: Two focus groups were conducted to explore participant experiences of ENHANCE. Data were analysed using inductive thematic analysis with constructs of the HAPA (motivational and volitional factors) as a framework. Results: We identified three themes (1) ‘The structured program addressed inactivity and improved feelings of wellness and preparation for the operation’. The benefits were not only physical, but psychological and were contextualised in terms of preparation for the upcoming surgery. (2) ‘People as enablers of participation’: Participants identified that the attitude, and skill of the experienced instructor were supportive and motivating, especially in tailoring the intervention. Within the program, the support of the group was considered a positive attribute (3) ‘Improved awareness changed attitudes to self-efficacy and perceived self-control’. Participants described an increased awareness of their condition and a better understanding of health expectations. They felt more control and ownership over their health journey. Conclusion: Goal setting and social support were identified factors in a behaviour-change counselling program, delivered in conjunction with structured exercise that led to a positive experience. Improved psychological and physical health were described. Participants were better prepared for their upcoming surgery, with increased self efficacy and mastery to support long-term physical-activity engagement.

## 1. Introduction

Osteoarthritis (OA), which can result in chronic pain and a major disability [1], is increasing in prevalence and may lead to surgery [2]. The recommendation of exercise in non-operative management is often forgotten despite international guidelines for managing OA recommending exercise as essential. Exercise can improve symptoms and the general well-being of people with OA without the risks of pharmacological treatments [3]. Specific exercise programs for patients with hip and knee OA have been shown to significantly reduce pain and improve function, performance and Quality of Life (QoL) [4,5]. Exercise is prescribed for symptomatic improvement once surgery is recommended for end-stage OA; however, this has shown equivocal results on function when implemented either before surgery or following surgery [6]. While people describe improvements in a range of patient’s reported outcomes after hip and knee arthroplasty, improvements in physical activity levels are not seen [7], and physical activity interventions delivered after surgery are not effective [8].

A comprehensive understanding of peoples’ belief about their pain is needed to provide patient-centred care for people living with knee or hip pain [9]. This requires us to delve into the perspectives of the people themselves, gather qualitative data of the reasons why physical activity may not improve once the pain from the joint is relieved with surgery [10]. To address the complex problem of the complications from low levels of physical activity that surgery itself does not improve, behaviour-change interventions that facilitate increases in physical activity and exercise are worth investigating.

The Health Action Process Approach (HAPA) [11] is a framework that provides structure to designing behaviour-change interventions. Changing behaviour involves two phases according to HAPA: The first is motivational, and includes risk perceptions, positive-outcome expectancies and action self-efficacy. These lead to an intention to change behaviour. The second phase is the doing, or volitional phase, that occurs after goal setting [12]. This volitional phase includes actional control and dealing with perceived barriers and benefits, and has elements of coping planning, recovery self-efficacy and maintenance self-efficacy, that result in the desired health behaviour. In this phase, people plan and undertake their exercise program, investing effort and recognizing that setbacks are inevitable, and learning strategies to overcome challenges for long-term success [12]. The recently published literature suggests that people with mild forms of osteoarthritis may see improvements in their action control with an HAPA based intervention [13].

This paper reports on the qualitative arm of a study that delivered a structured exercise program, including up to five in-person group counselling sessions, supported by written materials developed using the HAPA applied specifically to people with osteoarthritis. Using a thematic analysis [14] of focus-group data, the current study aimed to explore adults’ experiences of the 12-week behaviour-change and exercise program (ENHANCE).

## 2. Materials and Methods

### 2.1. Participants and Design

Participants on a wait list for hip or knee arthroplasty were recruited to take part in a 12-week randomised controlled trial to quantify the benefits of participating in a combined group-exercise training program and targeted behaviour-change counselling with the primary outcome of participation in physical activity compared to a control wait list of intervention. The comparison between the groups have been published separately [15]. The ENHANCE behaviour program for arthroplasty patients was led by an accredited exercise physiologist who delivered an individually tailored structured exercise program. The program also included up to five in-person counselling sessions that used researcher-developed written workshop materials to facilitate discussions within the group. Interaction between group members was facilitated so that the group members supported each other to encourage participation in physical activity. Participants were also encouraged to monitor their physical activity themselves. The protocol for the funded study has been previously published [16]. The study compared the intervention to a usual-care control group. Outcomes included daily physical activity (daily step count and percentage of day spent in sedentary activities) and pain ratings using a VAS. A number of self-reported functional outcomes and clinical markers linked with potential common chronic diseases were included as secondary outcomes. Constructs from HAPA [11] were included in the design of the behaviour-change intervention and included the provision of goal-setting, outcome expectancy, task self-efficacy, maintenance self-efficacy, planning and action. To be eligible for the study, participants were invited from a public surgery waitlist across one orthopedic clinic located in Launceston, Tasmania. Participants recruited undertook baseline assessments, and then were randomized to one of two groups by a researcher who was not involved in the data collection or intervention. For the purpose of evaluating the intervention, data were collected at baseline, at 26 weeks and at 26 weeks post-surgery.

Only participants from the exercise and behaviour-change counselling group were eligible for this study which aimed to explore adults’ experiences of the 12-week behaviour-change exercise program. The control group received usual care as recommended by evidence-based guidelines and a pamphlet from Exercise is Medicine explaining the benefits of exercise for adults with osteoarthritis [17]. Ethical approval was obtained. All and additional written consent required for participants attending the focus groups was obtained. Individuals who completed the exercise intervention were asked to participate in the focus groups.

### 2.2. ENHANCE Intervention

The exercise intervention was based on clinical evidence for hip and knee OA [18] and included aerobic, resistance and flexibility training components as previously described [16]. Programs were individualized based on each participant’s baseline assessment, co-morbidities and specific exercise needs by an accredited exercise physiologist and involved a combination of supervised group and home-based exercise. The group exercise sessions were supervised by an accredited exercise physiologist and involved 24 group-exercise sessions conducted twice a week over a 12-week period at the University of Tasmania’s Exercise Clinic. The home exercise programs which were completed unsupervised commenced in week 3 and were included to assist in their progression and confidence away from the exercise clinic. All programs were regularly reviewed and updated based on participants’ goals, participation in their home-based program, reported barriers and motivations for exercise, as well as reported symptoms and pain status.

The group sessions also involved an educational component at weeks 1, 2, 3, 6 and 12. The education sessions, guided by HAPA principles (see Table 1), involved the accredited exercise physiologist presenting and openly discussing with the group their experiences and knowledge of exercise.

Two focus groups were conducted and recorded. The number of included participants ranged from thee to six participants per group. This number of focus groups was selected based on previous research that outlines that 80% of themes can be elucidated from two to three groups. [19] The focus groups were facilitated by an experienced researcher (author JOB) who had not previously had any direct contact with the research participants. This female interviewer was an experienced exercise physiologist and nurse. Focus groups took place within six weeks of the exercise program finishing and were conducted at the University of Tasmania in an informal environment where participants were supported to feel comfortable, and light refreshments were provided. The participants were provided a $20 voucher as an expression of gratitude for their time to participate in the focus groups. All discussions were facilitated using a semi-structured interview guide (Table 2).

### 2.3. Data Analysis

Transcripts were coded and thematically analyzed in Microsoft Excel using a qualitative descriptive approach [14]. Two experienced researchers (authors MLB and JOB) familiarized themselves with the data, and then independently coded the transcripts inductively prior to generating a preliminary list of themes. The themes were then discussed with other available members of the wider group and agreed upon. Key quotes were selected from the transcripts to highlight the identified themes. Participants were given identification numbers related to their gender in the quotes, to give context and assure anonymity (e.g., PM1 is the first male participant and PF1 is the first female participant).

## 3. Results

### 3.1. Demographics

A total of 9 of the 29 participants who completed the exercise and behaviour-change intervention consented and took part in the focus groups (Female *n* = 5, Males *n* = 4, age mean 69.4 years, BMI 35.1 kg/m^2^). Demographic data (age, gender, surgery times and previous exercise history) were collected to contextualize the population. There were a range of co-morbidities that were shared across participants including Diabetes, Osteoarthritis, Cardiovascular Disease, Hypertension, neurological conditions and Asthma. The mean waiting time for surgery for this group was 358 (164) days, with a range from 179 to 618 days. Five participants completed high school as their highest level of formal education; two went to technical college; and two completed tertiary education studies to a post-graduate level.

Participant demographics are outlined in Table 3

We identified three themes. (1) ‘The structured program addressed inactivity and improved feelings of wellness and preparation for the operation’. Benefits were not only physical but psychological, and were contextualized in terms of preparation for the upcoming surgery. The HAPA volitional constructs of action and coping planning are reflected in this theme. Specifically, action planning was evident in terms of making plans on when, where how and with whom to exercise. Coping planning was also evident as part of the structure of the program, assisting participants to develop strategies to cope with barriers such as pain that may interfere with exercise. (2) ‘People as enablers of participation’. Participants identified that the attitude and skill of the experienced instructor were supportive and motivating, especially the tailoring of the intervention to meet individual needs. Within the program, the support of the group was considered a positive attribute and assisted with coping planning as participants were all managing a similar condition and sharing problem-solving strategies together. (3) ‘Improved awareness changed attitudes to self-efficacy and perceived self-control’. Participants described that through increased awareness of their condition they had a better understanding of health expectations and felt more control and ownership of their health journey, describing maintenance self-efficacy, recovery self-efficacy and action control.

#### 3.1.1. Theme One ‘Structured Program Addressed Inactivity and Improved Feelings of Wellness and Preparation for the Operation’

A common point raised by most of the participants is that they were inactive at the start of the program. While some had been active when younger, the development of joint degeneration had curbed their exercise and activity and they were quite sedentary:

*“I suppose like all people I played football and cricket when I was young. Did the right thing, got married and sport sort of faded away”* (PM2).

The group discussion was a positive influence on activity outside the program, as described by one participant:

*The second week I came, and we spoke about it, so I decided I’d do a little bit more at home, so each morning, I went through it [the exercises]* (PM1).

Almost all of the participants discussed a wide range of benefits that they perceived due to their involvement in the 12-week exercise program. Some of them were benefits to levels of activity: *“Yeah. So, I’ve virtually doubled what I used to do, it’s made it a lot stronger, a lot better.”* (PM4) and

*“–[i can] walk more which I couldn’t do before and it just made my life so much better and I think the exercise has worked terrifically.* (PF2). Whereas other benefits were related to holistic health like better sleep and reduced medication. For example

*“just be strengthening up around the knee. Before of a night my legs sort of rubbed together and after two or three hours I’d wake up in pain, but now I don’t, just because I’ve built them up.* (PM2), As well as: *‘… it did improve my knee a lot. Now I can go to bed of a night, I can sleep, and I don’t have to worry about taking Panadol.’ (PM3)* and *“it just made my life so much better”* (PF2).

Both the structure of the classes and individualization of the exercises were perceived positively by several participants. One participant reported that, *‘the whole thing was structured beautifully’* (PM3). Individualized exercise prescription was reported as follows: ‘*the fact that Mike took the time to assess each person and their capabilities and structured the exercises for you.*’ (PM3). Participants related that this individualization of exercises had two effects. Firstly, it set up a non-competitive vibe: *“… ‘everyone was individual, so we weren’t up against one another.’* (PF2) and ‘*we just went at our own pace, and we never compared with one another’* (PM2). Secondly, it meant that everyone could maximize their potential. For example *“there was this one lady that came that couldn’t even use the exercise bike and he just gave her little tips on what to do and did it gently and by the time we finished the 12 weeks, she was probably the best one on the exercise bike in the class.”* (PM1).

#### 3.1.2. Theme Two: People as Enablers of Participation

Participants described the importance of the skilled instructor in facilitating the sessions as a motivator for attendance. Additionally, the group context provided a supportive environment with ongoing friendships formed. The structure of the program designed by a qualified exercise physiologist that included individualized exercise within a group context was described as valuable. These data provided evidence of how both the experience and the attitude of the instructor were considered important, as described by one participant:

*……: What’s made it easier are two things, perhaps. The attitude of [instructor], and the program which has been structured.* (PF3).

Instructor skill and trust was especially important as the participants had come from a place of not exercising and, with significant clinical pathology, were unsure about embarking on an exercise program, as evidenced by the following comment:

*Because my hip hurts all the time but then I thought, ‘They should know what exercises to do for it,’ and they turned out all right anyway, so that was good…* (PF1).

This expertise gave participants confidence to continue their exercises: “*Well, it makes you confident that you’re doing the right thing by yourself, proves to yourself that you’re capable of doing it.* (PM1). *He knew our limitations, and that I think is very good for an instructor.* (PF5) *He did not leave us on our own, he was always there to guide you. He was very observant* (PF3).

As well, the instructor’s experience allowed for establishing a supportive environment. Two examples that demonstrate this follow: “*just to make you feel comfortable with what you’d done, and it was good for him to be able to tell you*”, (PM1) and ‘*The attitude of [the instructor], was so important. He has that, he encourages you to go on. He encourages you, he has your interest at heart, you know, and so for me I developed a love for the place, you know, for the exercises*.’ (PF5).

There were multiple described benefits including physical, social and mental benefits from being in a group and supporting each other. One example of the mental benefits is described as follows:

*‘getting with a group of people who are going through the same made a huge difference and not only the physical but the mental side of it’* (PF2). Ongoing social benefits were articulated; “*Yes, we–in our group, we became really close. We were going out for morning teas. We still ring each other up to keep in contact.*” (PF5) and “*I think we all kept an eye on one another*” (PM2) In fact, without the group, one person indicated that they were less likely to continue. ‘*I think when you go to a place to exercise and you take part in a group, you’re far more likely to do the exercises than if you do them at home on your own without equipment*.’ (PF3)

#### 3.1.3. Theme Three: Improved Awareness Changed Attitudes to Self-Efficacy and Perceived Self-Control

The way in which the participant engaged with the exercise program changed over the course of the program in concordance with self-management principles; this was evident in participants recognizing improved awareness of the benefit of exercise and accountability for their future selves using mental imagery. An example of this is the comment

*“Doing this course [the Enhance] was really a great help. It’s really got me aware of how important it [exercise] is and how I feel stronger”* (PF2).

There was an ongoing effect to function, as described by one participant: “*I can walk up and down stairs -**…**- I’m finding I can manage the stairs. I couldn’t before and shopping I can do now which I couldn’t.* Concomitant to the improvement in function came a sense of mastery and independence: *“So I just got into it and really enjoyed it, to the point where I do the same program at home now*”, (PM2) and “*Well, I bought myself a little pedal bike”* (PF4).

Goal setting was linked to formulating the intention to exercise, assisted participants to become aware of their intrinsic motivation and to understand their perceived control of the situation.

*‘You’d set yourself goals…… You try to improve each time.’* (PM1).

Participants developed confidence over time to maintain their regular exercise despite barriers and took the time to reflect on what had worked in the past for them; thereby, improved mastery had an ongoing effect on confidence or recovery self-efficacy.

*“Well, it makes you confident that you’re doing the right thing by yourself, proves to yourself that you’re capable of doing it. making an effort to improve what’s going to happen to you. I think it’s just your self-motivation”* (PM1).

## 4. Discussion

The results of our study show that an individualized group-based exercise and behaviour-change program for adults with osteoarthritis has multifaceted benefits for participants. Our findings add to the body of literature on adults waiting for hip and knee surgery–a population which is often not included in research trials. Our results showed perceived improvements in components of physical health such as strength, and in psychological health, through social support. Structured and well-facilitated sessions were important for ENHANCE participants. Participants also developed a sense of mastery which may be helpful for sustaining new habits in their physical-activity behaviours.

Research investigating changes in activity for people on wait lists for joint replacement is challenging, with several recent randomised controlled outcome-focused studies unable to recruit sufficient sample sizes to identify quantitative changes in activity after surgery [15,20]. Reasons described in the literature include the challenges from a time and distance perspective in attending face-to-face exercise and behaviour-change interventions [15,20]. To better understand the perspectives of people with lived experience of osteoarthritis while waiting for joint replacement, qualitative studies such as this are needed.

Goal setting and social-support elements of our behaviour-change counselling program described in these themes, are linked to physical-activity engagement The data in the current study adds to the previously published literature, where social support is identified as a strong motivator for change and increasing adherence to physical activity. [10,21] Social support is also one of the frequently reported modifiable behaviour supports for improving engagement in self-management [22]. Patient-led goal setting, self-monitoring of behaviour and social support have previously been highly effective in promoting physical-activity adherence in chronic health conditions [23], and specifically in an OA population [10]. As our program used a theoretical foundation (HAPA), the elements of action planning and action control are evidenced in the data from the participants. Action planning and action control are supported by the other literature investigating musculoskeletal pain that indicates goal setting is an effective component of behaviour-change interventions in that population. [24] Participants developed a sense of mastery which may be helpful for sustaining new habits in their physical-activity behaviours. An increased awareness of the importance of exercise led to actual changes in behaviour, with participants describing increased physical activity (exercise) and ongoing effects to wellbeing. Other programs looking to develop long-term changes in attitude to physical activity in a sedentary clinical population can consider using action planning and action control in their study design.

Attitude to exercise is important. Our data indicate that a change in awareness and increased self-efficacy to exercise was a driver for increasing engagement in physical activity. While evidence on the effectiveness of education alone on improving self-efficacy is inconclusive, [25] our program that included a behaviour-change component was more successful in producing attitudinal shifts favoring physical activity. Instructors were able to provide feedback on performance and changes overtime, which has been shown to improve physical activity after joint replacement. One mechanism by which feedback is shown to change behaviour is suggested in the literature to be by improving self-efficacy [26]. Self-efficacy has been reported to be useful for problem solving, to overcome barriers to physical activity [21,27]. Increasing self-efficacy can overcome the multiple physical-activity barriers that people with OA have, and can highlight the benefits of increasing self-efficacy [28] in programs designed to improve long-term physical activity.

Our program ‘ENHANCE’ combined instructor-led exercises and behaviour-change-counselling techniques whilst coaching participants to use their own exercise booklet to progress from understanding the benefits of exercise, to planning on when, where and how to perform the exercises, through to preparing for setbacks and imagining the benefits of exercising post-surgery. Previous research indicates that support and motivation from healthcare workers are important factors in facilitating exercise participation in people with hip and knee OA [28]. This was also reflected in the current study, whereby trust in the instructor was crucial to success. Health professionals have an important role in empowering their clients to use a range of behaviour-change techniques to make positive health decisions, such as improving physical activity engagement. Both physical and psychological benefits were seen in the current study, adding to the literature which indicates these two constructs are interwoven in people with hip and knee osteoarthritis [29].

This study has several limitations. These findings are relevant to the context of health care in our country. Furthermore, while the qualitative nature of the study design allowed for in-depth investigation of the perspectives of the participants, the number of participants was small which may limit the transferability of the results.

## 5. Conclusions

This study demonstrated that a structured, instructor-led group-based exercise and behaviour-change counselling program based on HAPA was beneficial from multiple perspectives for adults with OA. Improved psychological and physical health was described. Changes in attitude towards physical-activity engagement was underpinned by improved self efficacy and mastery, with social support added to the benefits. Thus, health professionals can consider including behaviour-change techniques in their practice, which may enhance the effectiveness of interventions; therefore, promoting physical activity to inactive adults on the hip and knee waitlist for surgery.

## Figures and Tables

**Table 1:** Constructs of behaviour change delivered throughout the ENHANCE intervention.

Intervention Components	Behaviour Change Techniques	Potential Mediators
Motivational component.		
Providing information on the risk factors of sedentary lifestyle.	Information provision Mental imagery	Risk perception
Providing information on the benefits and advantages of regular walking.	Information provision Mental imagery	Positive-outcome expectancy
Establishing confidence to start regular walking.	Resources identification Modelling (modelling by others) Mental imagery Verbal persuasion	Action self-efficacy
Formulating the intentions of regular walking.	Intention formation Goal setting	Intention
Volitional component.		
Making plans on when, where, how, and with whom to conduct regular walking.	Planning exercise	Action planning
Developing strategies to cope with the barriers that may interfere with regular walking.	Barriers identification Problem solving	Coping planning
Developing confidence of maintaining regular walking with barriers, as well as resuming regular walking behaviours if interrupted.	Mental imagery Mastery experience (past experience)	Maintenance self-efficacy Recovery self-efficacy
Developing strategies to remind and monitor regular walking.	Self-monitoring exercise Reminders and sign-in table	Action control

**Table 2:** Guiding questions utilized during focus groups.

Category	Questions and Probes
Background	Can you tell me a little bit about your previous experience with exercise? Can you tell me your previous experience of being in group exercises or a gym setting?
Expectations and interest in participation	Tell me your reasons for wanting to take part in the ENHANCE program? Probe: What did you initially expect from the ENHANCE program? On a scale of 1 to 5 (1 being the lowest and 5 being the highest rating), how well were your expectations met? Probe: Can you explain your reasons for selecting this? Tell me how you felt about the study/exercise as you were in the waiting room for the first time. Tell me how you felt when you were told that you were going to be in the intervention group. How would you say your thoughts changed throughout the 12 weeks?
Education sessions	As part of the ENHANCE program you participated in group education sessions with an accredited exercise physiologist. During these sessions, you set goals and developed habits to change exercise and physical-activity behaviours. What was it like to participate in group education sessions with this health professional? Probe to identify likes, dislikes, suggested changes. Probe to compare this experience to a consulting health professional (e.g., GP or nurse) online? Tell me about the advice you received. Probe to identify the degree to which their needs, goals, barriers, tailored the advice based on their needs and expectations. Probe to expand/clarify response by probing on motivation, encouragement. Tell me your thoughts about the amount of contact you had with the group education sessions? Prompt: Frequency–how many group sessions did you attend and your reasons for attending or not? Duration–were the sessions long enough? How was the homework component? Would you have liked to do this as a group? Timing–did this work? Would online work? Since being involved in ENHANCE, what have your exercise/PA behaviours been like? Prompts: If your physical activity behaviours have not changed, what are reasons they did not change? Prompts: What plans, if any, do you have to maintain any changes?
Outcomes of the program	Did you notice any differences in yourself?
Barriers and facilitators	Which things made it easier for you to participate, and which things made it harder?

**Table 3:** Characteristics of the Study Participants.

Id. No.	Gender	Age	BMI	Conditions	Education	Exercise Last 7 Days	Total Days Awaiting Surgery
PF1	F	71	30.5	Diabetes, Osteoarthritis	Post grad	0	337
PM1	M	70	29.4	Cardiovascular Disease, Osteoarthritis	Technical college	0	397
PF2	F	78	43.2	Cardiovascular Disease			243
PM2	M	75	30.1	Asthma, Osteoarthritis	Yr10	0	179
PF3	F	65	40	High Blood Pressure, Cardiovascular Disease, Osteoarthritis	Technical college	7	225
PF4	F	70	31.2	Osteoarthritis, Cancer	Post grad	7	235
PM3	M	69	30.5	High Blood Pressure, Osteoarthritis	Yr10	2	530
PF5	F	59	48.3	Diabetes, High Blood Pressure, Arthritis	Yr10	3	618
PM4	M	68	32.7	High Blood Pressure, Arthritis	Yr12	0	552

PF = participant female, PM = participant male.

## Data Availability

Data are available from the corresponding author on reasonable request.
